# *mer*-Tri­chlorido­tris­(tetra­hydro­thio­phene-κ*S*)iridium(III): preparation and comparison with other *mer*-tri­chlorido­tris­(tetra­hydro­thio­phene-κ*S*)metal complexes

**DOI:** 10.1107/S2056989016012883

**Published:** 2016-08-16

**Authors:** Loren C. Brown, Christine M. DuChane, Joseph S. Merola

**Affiliations:** a Department of Chemistry, Virginia Tech, Blacksburg, VA 24061, USA

**Keywords:** crystal structure, iridium, tetra­hydro­thio­phene, conformers

## Abstract

The title complex, [IrCl_3_(C_4_H_8_S)_3_], was prepared according to a literature method. A suitable crystal was obtained by diffusion of pentane into a di­chloro­methane solution and analyzed by single-crystal X-ray diffraction at 100 K. The title complex is isotypic with *mer*-tri­chlorido­tris­(tetra­hydro­thio­phene-κ*S*)rhodium(III). However, the orientation of the tetra­hydro­thio­phene rings is different from an earlier report of *mer*-tri­chlorido­tris­(tetra­hydro­thio­phene-κ*S*)iridium(III) deposited in the Cambridge Structural Database. The IrS_3_Cl_3_ core shows a nearly octa­hedral structure with various bond angles within 1–2° of the perfect 90 or 180° expected for an octa­hedron. The structure of the title compound is compared with the previous iridium complex as well as the rhodium and other octa­hedral metal tris-tetra­hydro­thio­phene compounds previously structurally characterized. DFT calculations were performed, which indicate the *mer* isomer is significantly lower in energy than the *fac* isomer by 50.1 kJ mol^−1^, thereby accounting for all compounds in the CSD being of the *mer* geometry. Powder X-ray diffraction of the bulk material showed that the preparation method yielded only the isomorph reported in this communication.

## Abstract 2

The crystal structure of *mer*-tri­chlorido­tris­(tetra­hydro­thio­phene-κ*S*)iridium(III) is reported and compared with a different form of the complex previously reported. It is also compared with other *mer*-tri­chlorido­tris­(tetra­hydro­thio­phene-κ*S*)metal(III) complexes of molybdenum, ruthenium and rhodium.

## Chemical context   

We have been engaged in various studies of iridium chemistry for many years (Merola, 1997 ▸; Merola & Franks, 2015 ▸; Merola *et al.*, 2013 ▸) and recently had need to find alternate routes to some iridium(III) complexes for our research. An examination of the literature led to the title compound as a possible anhydrous source of iridium(III) that we could use as a starting material (Allen & Wilkinson, 1972 ▸). *mer*-Tri­chlorido­tris­(tetra­hydro­thio­phene-κ*S*)iridium(III) has been mentioned in the literature as a starting material for other organometallic iridium complexes (Hay-Motherwell *et al.*, 1989 ▸, 1992 ▸, 1990 ▸; John *et al.*, 2000 ▸, 2001 ▸, 2014 ▸), and most recently has been the starting material of choice for new emissive materials (Chang *et al.*, 2008 ▸, 2011 ▸, 2013 ▸; Chiu *et al.*, 2009 ▸; Hung *et al.*, 2010 ▸; Lin, Chang *et al.*, 2011 ▸; Lin, Chi *et al.*, 2011 ▸; Lin *et al.*, 2012 ▸). However, no crystallographic studies had been published on this compound. Given its increasing importance, we decided that a single crystal structure determination of the title compound would be worthwhile.

## Structural commentary   

**Figure 1:**
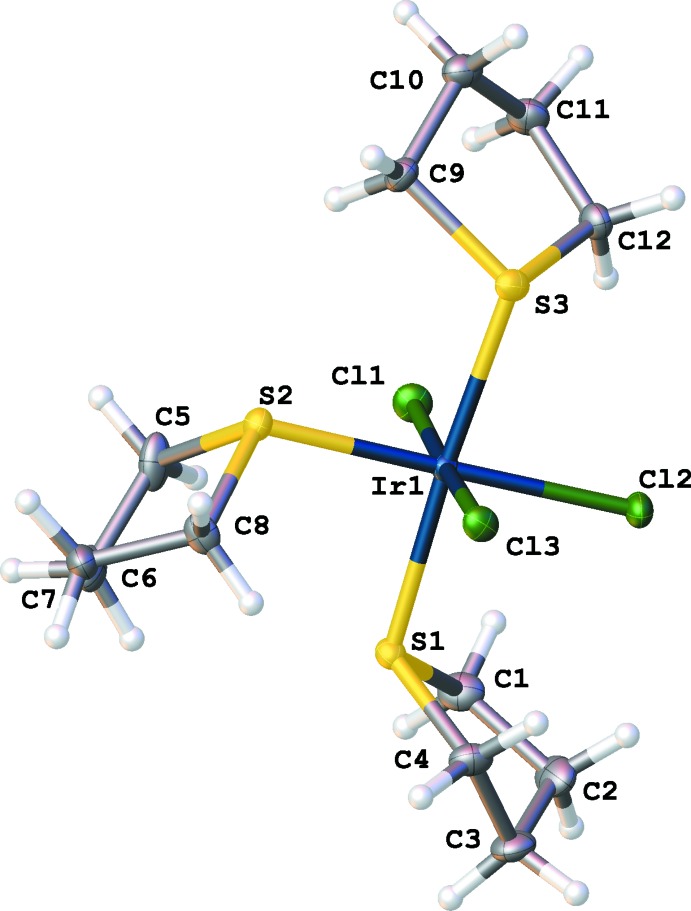
Displacement ellipsoid plot (50% probability) of *mer*-tri­chlorido­tris(tetra­hydro­thio­phene-κ*S*)iridium(III) (CCDC 1495966).

*mer*-Tri­chlorido­tris­(tetra­hydro­thio­phene-κ*S*)iridium(III) (CCDC refcode 1495966) crystallizes in the *P*2_1_/*n* space group with one mol­ecule in the asymmetric unit (Fig. 1 ▸). The core structure (heavy atoms around the iridium) is very close to rigorous octa­hedral geometry with the largest angular variation [Cl1—Ir1—Cl33, 177.35 (3)°] being less than 2.7° from ideal linearity.
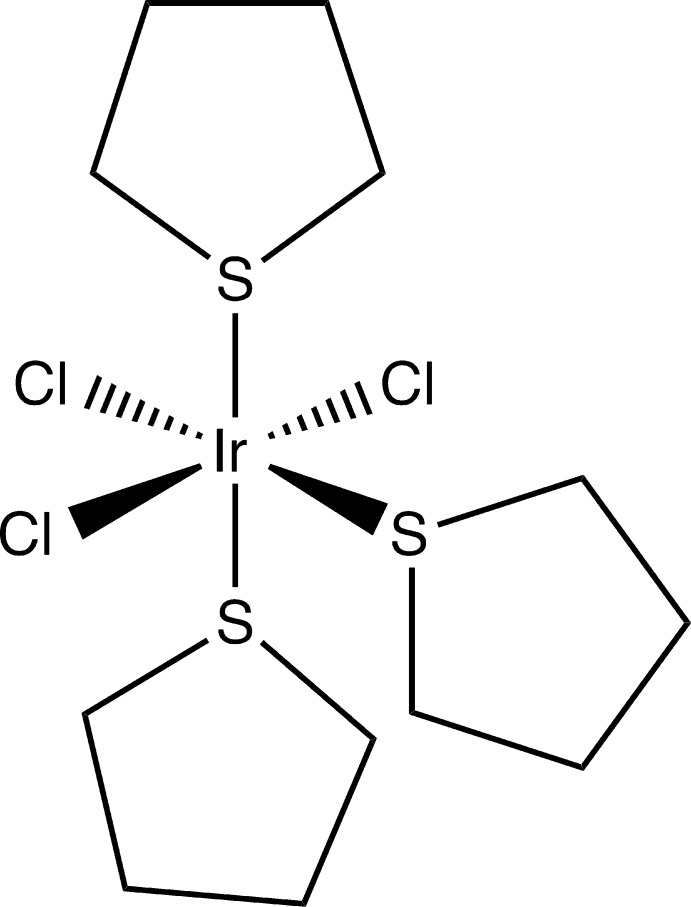


 The Ir—Cl bond lengths [range 2.3648 (8)–2.3774 (9) Å] are somewhat longer than the Ir—S bonds [range 2.3279 (9)–2.3575 (9) Å], as expected from the slightly larger radius of Cl. A search for Ir—S bonds in the CSD (Groom *et al.*, 2016 ▸) and analyzed with *Mercury* (Macrae *et al.*, 2008 ▸) found 2566 instances with distances ranging from 2.134 to 2.633 Å and a mean value of 2.358 Å. That places the bond lengths for the title compound slightly above the mean value. Similarly, a *Mercury* data analysis of the CSD for Ir—Cl bond lengths found 3965 instances with distances ranging from 2.121 to 2.816 Å and a mean value of 2.413 Å, which places the Ir—Cl distances for the title compound lower than the mean. This comparison should not be considered as too significant since it was not possible to compare bond lengths only for iridium(III) compounds and the analysis includes quite a few iridium(I) complexes. The tetra­hydro­thio­phene rings are well ordered in the title structure, adopting a puckered conformation consistent with trying to minimize ring strain. Two of the rings are positioned with the center of the ring aligned over a chlorine atom in the structure, while the third is aligned over a sulfur atom of another ring. More will be said about the ring conformations in the *Database survey* section.

## Supra­molecular features   

An examination of the packing diagrams for the title compound shows no unusual inter­molecular features other than van der Waals inter­actions.

## Database survey   

**Figure 2:**
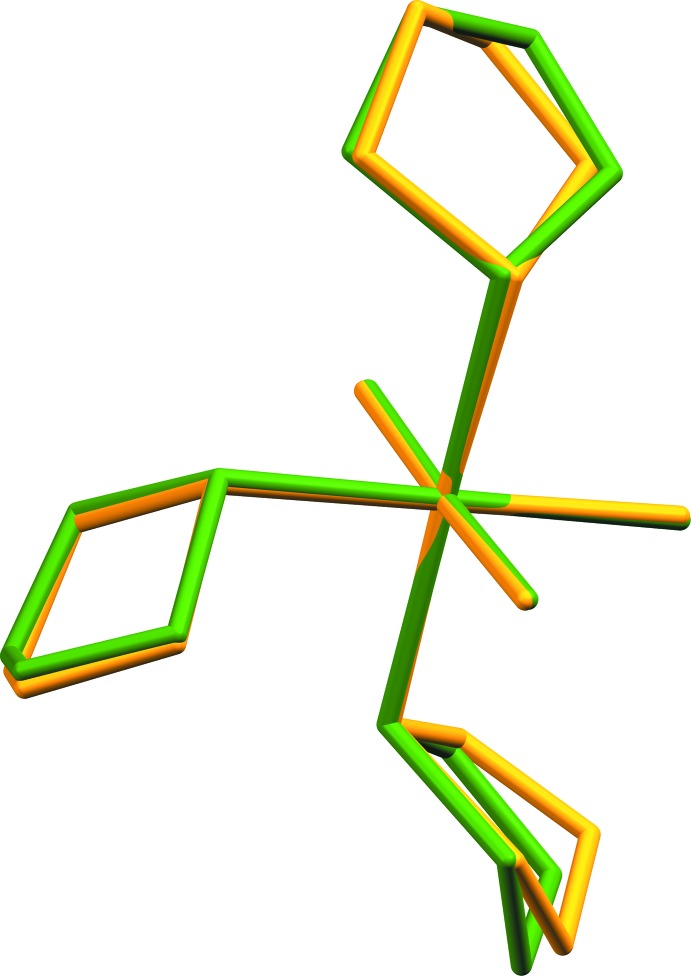
Calculated overlay of two polymorphs of *mer*-tri­chlorido­tris­(tetra­hydro­thio­phene-κ*S*)iridium(III) (CCDC 1438699 and CCDC 1495966). Structure from this paper shown in yellow.

**Figure 3:**
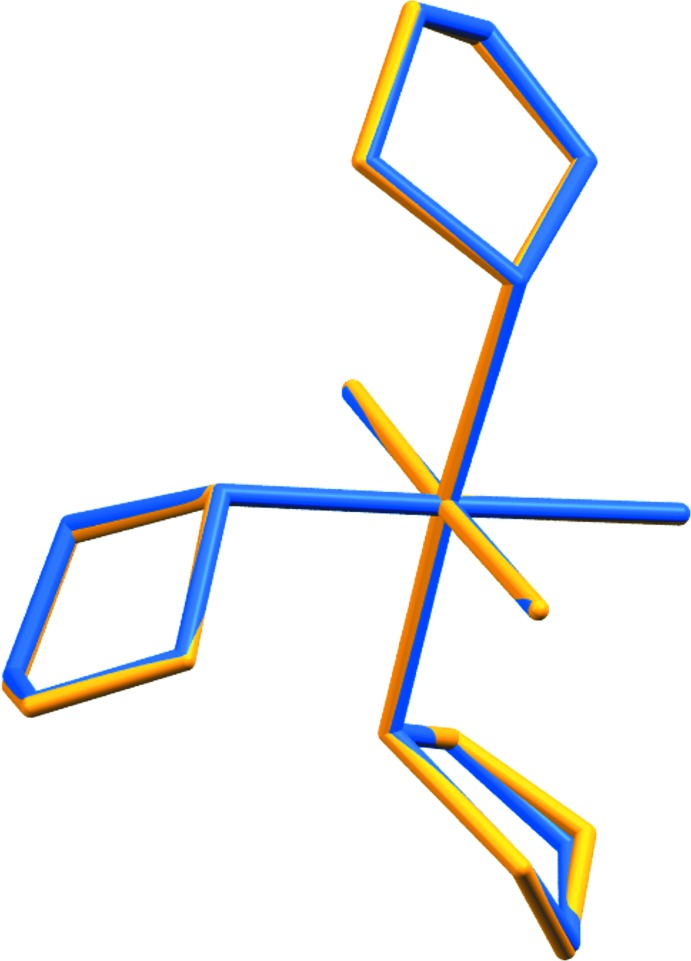
Calculated overlay of *mer*-tri­chlorido­tris­(tetra­hydro­thio­phene-κ*S*)iridium(III) (CCDC 1495966) in yellow with the isotypical rhodium complex (CCDC GEZHUO) in blue.

A survey of the CCDC database (Groom *et al.*, 2016 ▸) uncovered a number of metal *mer*-tris­(THT-κ*S*)metal complexes (THT= tetra­hydro­thio­phene), including one iridium structure deposited as a private communication (CCDC 1438699; Rheingold & Donovan-Merkert, 2015 ▸). The deposited structure (CCDC 1438699) packs with very different unit-cell parameters but the overall mol­ecular structure is substanti­ally the same. The results of the different packing, however, are slightly different conformations of two of the three THT ligands, as shown in Fig. 2 ▸, a structure overlay calculated in *Mercury* (Macrae *et al.*, 2008 ▸). On the other hand, the rhodium(III) complex is isotypic with the title complex with similar unit-cell parameters (CCDC refcode GEZHUO; Clark *et al.*, 1988 ▸). Fig. 3 ▸ shows an overlay calculated with *Mercury* (Macrae *et al.*, 2008 ▸) of the title complex with the rhodium compound, showing the nearly perfect atomic overlay. Ruthenium(III) (VIJYAO; Yapp *et al.*, 1990 ▸) and molybdenum(III) (REDXIH; Boorman *et al.*, 1996 ▸) complexes were also found in the database, with all showing the same meridional arrangement of ligands with the exception that the ruthenium complex displays disorder from overlapping conformations of one of the THT ligands.

## Theoretical calculations   

**Figure 4:**
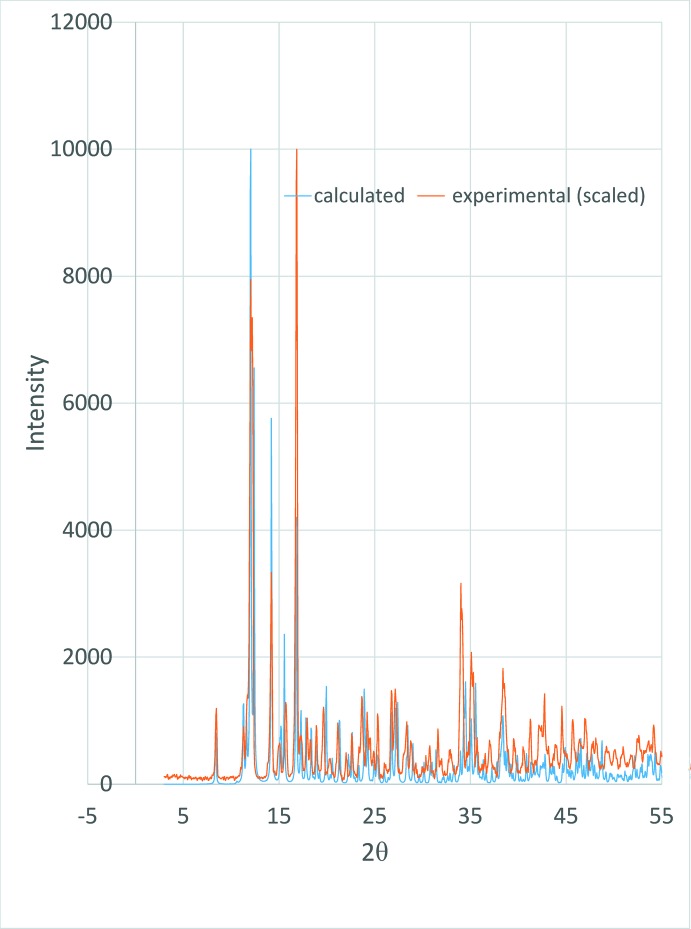
Powder X-ray diffraction pattern of title compound collected on a Rigaku Miniflex 600 Powder X-ray diffractometer compared with pattern simulated by *Mercury* (Macrae *et al.*, 2008 ▸). Experimental and simulated patterns scaled to highest intensity peak in each.

We were inter­ested in determining if the bulk material synthesized by this process is of a single polymorph or if both of the iridium structures reported (CCDC 1495966, this report, and CCDC 1438699, Rheingold & Donovan-Merkert, 2015 ▸) were present. Fig. 4 ▸ shows an overlay of the powder X-ray diffraction pattern for the complex reported here with the powder pattern predicted by *Mercury* (Macrae *et al.*, 2008 ▸). The match is very good and quite distinct from the pattern predicted for CCDC 1438699, indicating that the bulk material formed in this process is a single polymorph matching the structure reported here.

One feature that stands out in all cases is that the *M*Cl_3_(THT)_3_ compounds found in the database adopt the *mer* configuration. Calculations were performed using density functional theory with *Gaussian 09* (Frisch *et al.*, 2009 ▸). Full geometry optimization of both the *mer* and *fac* isomers was carried out *via* density functional theory (DFT) with the Becke-3-parameter exchange functional (Becke, 1993 ▸) and the Lee–Yang–Parr correlation functional (Lee *et al.*, 1988 ▸). Because iridium is not covered in the cc-PVDZ basis set used, computations involving Ir employed Stuttgart/Dresden quasi-relativistic pseudopotentials (Andrae *et al.*, 1990 ▸). The difference between the two isomers was quite large with the *mer* isomer being more stable than the *fac* by 50.1 kJ mol^−1^, suggesting the occurrence of only the *mer* isomer for the small set of compounds surveyed may be due to thermodynamic stability.

## Synthesis and crystallization   

The title compound was synthesized using a slight modification of a literature procedure (John *et al.*, 2014 ▸). IrCl_3_·3H_2_O (1.00 g, 2.84 mmol) and 2-meth­oxy­ethanol (50 mL) were added to a 250 mL round-bottomed flask fitted with a magnetic stir bar and a reflux condenser. Tetra­hydro­thio­phene (1.25 mL, 14.2 mmol) was added all at once with stirring. The resulting suspension was refluxed for 18 h, providing a clear orange solution that gave a yellow precipitate upon cooling to room temperature. Deionized water (75 mL) was added and the suspension was cooled overnight (273 K) before collection on a fine-porosity sintered glass frit. The resulting yellow powder was washed with deionized water (3 x 15 mL) then cold ethanol (3 x 15 mL). After vacuum drying overnight the yellow powder (1.40 g, 88%) was characterized by ^1^H and ^13^C NMR spectroscopy. As the NMR spectra were in agreement with previously reported data, no further purification was necessary. Single crystals for X-ray diffraction were grown by slow diffusion of *n*-pentane into a di­chloro­methane solution of *mer*-IrCl_3_(THT)_3_.

## Refinement   

Crystal data, data collection and structure refinement details are summarized in Table 1 ▸.

## Supplementary Material

Crystal structure: contains datablock(s) I. DOI: 10.1107/S2056989016012883/pk2588sup1.cif

Structure factors: contains datablock(s) I. DOI: 10.1107/S2056989016012883/pk2588Isup2.hkl

Supporting information file. DOI: 10.1107/S2056989016012883/pk2588Isup3.mol

CCDC reference: 1495966

Additional supporting information: crystallographic information; 3D view; checkCIF report

## Figures and Tables

**Table 1:** Experimental details

Crystal data	
Chemical formula	[IrCl_3_(C_4_H_8_S)_3_]
*M*_r_	563.04
Crystal system, space group	Monoclinic, *P*2_1_/*n*
Temperature (K)	100
*a*, *b*, *c* (Å)	11.9160 (3), 10.2528 (2), 14.9434 (4)
β (°)	107.202 (3)
*V* (Å^3^)	1744.00 (7)
*Z*	4
Radiation type	Mo *K*α
μ (mm^−1^)	8.46
Crystal size (mm)	0.51 × 0.43 × 0.32

Data collection	
Diffractometer	Rigaku OD Xcalibur Eos Gemini ultra
Absorption correction	Analytical [*CrysAlis PRO* (Rigaku Oxford Diffraction, 2015 ▸) based on expressions derived by Clark & Reid (1995 ▸)]
*T*_min_, *T*_max_	0.064, 0.155
No. of measured, independent and observed [*I* > 2σ(*I*)] reflections	19537, 5773, 5062
*R*_int_	0.042
(sin θ/λ)_max_ (Å^−1^)	0.751

Refinement	
*R*[*F* ^2^ > 2σ(*F* ^2^)], *wR*(*F* ^2^), *S*	0.029, 0.063, 1.05
No. of reflections	5773
No. of parameters	172
H-atom treatment	H-atom parameters constrained
Δρ_max_, Δρ_min_ (e Å^−3^)	1.54, −1.46

Computer programs: *CrysAlis PRO* (Rigaku Oxford Diffraction, 2015 ▸), *SHELXT* (Sheldrick, 2015*a* ▸), *SHELXL2016* (Sheldrick, 2015*b* ▸), *OLEX2* (Dolomanov *et al.*, 2009 ▸) and *Mercury* (Macrae *et al.*, 2008 ▸).

## supplementary crystallographic information

### Crystal data

**Table:**

[IrCl_3_(C_4_H_8_S)_3_]	*F*(000) = 1088
*M**_r_* = 563.04	*D*_x_ = 2.144 Mg m^−^^3^
Monoclinic, *P*2_1_/*n*	Mo *K*α radiation, λ = 0.71073 Å
*a* = 11.9160 (3) Å	Cell parameters from 9679 reflections
*b* = 10.2528 (2) Å	θ = 4.1–32.2°
*c* = 14.9434 (4) Å	µ = 8.46 mm^−^^1^
β = 107.202 (3)°	*T* = 100 K
*V* = 1744.00 (7) Å^3^	Cube, yellow
*Z* = 4	0.51 × 0.43 × 0.32 mm

### Data collection

**Table:**

Rigaku OD Xcalibur Eos Gemini ultra diffractometer	5773 independent reflections
Radiation source: fine-focus sealed X-ray tube, Enhance (Mo) X-ray Source	5062 reflections with *I* > 2σ(*I*)
Graphite monochromator	*R*_int_ = 0.042
Detector resolution: 8.0061 pixels mm^-1^	θ_max_ = 32.2°, θ_min_ = 3.6°
ω scans	*h* = −13→17
Absorption correction: analytical [CrysAlis PRO (Rigaku Oxford Diffraction, 2015) based on expressions derived by Clark & Reid (1995)]	*k* = −13→15
*T*_min_ = 0.064, *T*_max_ = 0.155	*l* = −22→21
19537 measured reflections	

### Refinement

**Table:**

Refinement on *F*^2^	Primary atom site location: structure-invariant direct methods
Least-squares matrix: full	Hydrogen site location: inferred from neighbouring sites
*R*[*F*^2^ > 2σ(*F*^2^)] = 0.029	H-atom parameters constrained
*wR*(*F*^2^) = 0.063	*w* = 1/[σ^2^(*F*_o_^2^) + (0.0223*P*)^2^ + 0.8135*P*] where *P* = (*F*_o_^2^ + 2*F*_c_^2^)/3
*S* = 1.05	(Δ/σ)_max_ = 0.002
5773 reflections	Δρ_max_ = 1.54 e Å^−^^3^
172 parameters	Δρ_min_ = −1.46 e Å^−^^3^
0 restraints	

### Special details

**Table:**

Geometry. All esds (except the esd in the dihedral angle between two l.s. planes) are estimated using the full covariance matrix. The cell esds are taken into account individually in the estimation of esds in distances, angles and torsion angles; correlations between esds in cell parameters are only used when they are defined by crystal symmetry. An approximate (isotropic) treatment of cell esds is used for estimating esds involving l.s. planes.

### Fractional atomic coordinates and isotropic or equivalent isotropic displacement parameters (Å^2^)

**Table:**

	*x*	*y*	*z*	*U*_iso_*/*U*_eq_	
Ir1	0.45092 (2)	0.70407 (2)	0.73222 (2)	0.01133 (4)	
Cl1	0.59718 (7)	0.70422 (8)	0.65431 (6)	0.01922 (17)	
Cl2	0.59716 (8)	0.73216 (8)	0.87865 (6)	0.01887 (16)	
Cl3	0.30344 (7)	0.71455 (8)	0.80946 (6)	0.01688 (16)	
S1	0.46678 (7)	0.47667 (8)	0.74921 (6)	0.01509 (16)	
S2	0.30373 (7)	0.68096 (8)	0.59053 (6)	0.01540 (16)	
S3	0.44035 (7)	0.93361 (9)	0.72529 (6)	0.01636 (16)	
C1	0.6230 (3)	0.4314 (4)	0.7907 (3)	0.0236 (8)	
H1A	0.6388	0.3570	0.7540	0.028*	
H1B	0.6727	0.5059	0.7837	0.028*	
C2	0.6496 (3)	0.3940 (4)	0.8934 (3)	0.0281 (9)	
H2A	0.7157	0.3314	0.9114	0.034*	
H2B	0.6710	0.4722	0.9337	0.034*	
C3	0.5384 (4)	0.3321 (4)	0.9044 (3)	0.0257 (8)	
H3A	0.5449	0.3203	0.9715	0.031*	
H3B	0.5246	0.2459	0.8732	0.031*	
C4	0.4380 (3)	0.4262 (4)	0.8582 (3)	0.0216 (7)	
H4A	0.4387	0.5024	0.8991	0.026*	
H4B	0.3610	0.3820	0.8448	0.026*	
C5	0.3455 (3)	0.5674 (4)	0.5104 (3)	0.0277 (9)	
H5A	0.3271	0.6057	0.4469	0.033*	
H5B	0.4308	0.5488	0.5331	0.033*	
C6	0.2749 (3)	0.4419 (4)	0.5082 (3)	0.0261 (8)	
H6A	0.2620	0.3969	0.4474	0.031*	
H6B	0.3179	0.3822	0.5588	0.031*	
C7	0.1582 (3)	0.4802 (4)	0.5219 (3)	0.0225 (8)	
H7A	0.1078	0.5244	0.4654	0.027*	
H7B	0.1163	0.4024	0.5348	0.027*	
C8	0.1883 (3)	0.5720 (4)	0.6051 (3)	0.0192 (7)	
H8A	0.2167	0.5224	0.6643	0.023*	
H8B	0.1182	0.6229	0.6065	0.023*	
C9	0.3843 (3)	0.9968 (4)	0.6060 (2)	0.0188 (7)	
H9A	0.3912	0.9305	0.5597	0.023*	
H9B	0.3010	1.0232	0.5920	0.023*	
C10	0.4624 (3)	1.1147 (4)	0.6047 (3)	0.0213 (7)	
H10A	0.4527	1.1433	0.5396	0.026*	
H10B	0.4424	1.1883	0.6400	0.026*	
C11	0.5875 (3)	1.0686 (4)	0.6510 (3)	0.0228 (8)	
H11A	0.6424	1.1434	0.6631	0.027*	
H11B	0.6119	1.0058	0.6099	0.027*	
C12	0.5882 (3)	1.0027 (4)	0.7436 (3)	0.0209 (7)	
H12A	0.6061	1.0674	0.7951	0.025*	
H12B	0.6483	0.9330	0.7598	0.025*	

### Atomic displacement parameters (Å^2^)

**Table:**

	*U*^11^	*U*^22^	*U*^33^	*U*^12^	*U*^13^	*U*^23^
Ir1	0.01159 (6)	0.01222 (7)	0.01049 (6)	0.00034 (4)	0.00376 (5)	−0.00076 (4)
Cl1	0.0165 (4)	0.0225 (4)	0.0222 (4)	−0.0003 (3)	0.0112 (3)	−0.0008 (3)
Cl2	0.0199 (4)	0.0171 (4)	0.0157 (4)	−0.0013 (3)	−0.0007 (3)	−0.0021 (3)
Cl3	0.0183 (4)	0.0179 (4)	0.0177 (4)	0.0004 (3)	0.0102 (3)	−0.0020 (3)
S1	0.0158 (4)	0.0132 (4)	0.0156 (4)	0.0007 (3)	0.0035 (3)	−0.0012 (3)
S2	0.0151 (4)	0.0170 (4)	0.0130 (4)	0.0001 (3)	0.0024 (3)	0.0001 (3)
S3	0.0202 (4)	0.0152 (4)	0.0157 (4)	0.0008 (3)	0.0084 (3)	−0.0004 (3)
C1	0.0173 (17)	0.0225 (18)	0.032 (2)	0.0057 (14)	0.0092 (16)	0.0040 (16)
C2	0.0209 (18)	0.025 (2)	0.030 (2)	0.0032 (15)	−0.0046 (17)	0.0039 (17)
C3	0.031 (2)	0.0182 (18)	0.026 (2)	0.0064 (15)	0.0056 (17)	0.0081 (16)
C4	0.0242 (18)	0.0193 (18)	0.0240 (18)	0.0017 (14)	0.0113 (16)	0.0053 (15)
C5	0.0195 (18)	0.046 (3)	0.0179 (18)	0.0012 (17)	0.0059 (15)	−0.0126 (17)
C6	0.032 (2)	0.0249 (19)	0.0184 (18)	0.0067 (16)	0.0027 (16)	−0.0076 (16)
C7	0.0290 (19)	0.0168 (17)	0.0180 (17)	−0.0032 (14)	0.0015 (16)	−0.0019 (14)
C8	0.0143 (15)	0.0214 (17)	0.0238 (18)	−0.0031 (13)	0.0082 (14)	−0.0051 (15)
C9	0.0191 (17)	0.0207 (17)	0.0149 (16)	0.0011 (13)	0.0023 (14)	0.0031 (13)
C10	0.0262 (18)	0.0181 (17)	0.0212 (18)	0.0021 (14)	0.0097 (16)	0.0024 (15)
C11	0.0222 (18)	0.0221 (18)	0.0260 (19)	−0.0030 (14)	0.0100 (16)	0.0021 (15)
C12	0.0189 (17)	0.0199 (18)	0.0209 (18)	−0.0037 (13)	0.0013 (15)	−0.0008 (14)

### Geometric parameters (Å, º)

**Table:**

Ir1—Cl1	2.3648 (8)	C5—H5A	0.9900
Ir1—Cl2	2.3774 (9)	C5—H5B	0.9900
Ir1—Cl3	2.3732 (8)	C5—C6	1.533 (6)
Ir1—S1	2.3469 (9)	C6—H6A	0.9900
Ir1—S2	2.3279 (9)	C6—H6B	0.9900
Ir1—S3	2.3575 (9)	C6—C7	1.516 (5)
S1—C1	1.839 (4)	C7—H7A	0.9900
S1—C4	1.835 (4)	C7—H7B	0.9900
S2—C5	1.841 (4)	C7—C8	1.515 (5)
S2—C8	1.834 (3)	C8—H8A	0.9900
S3—C9	1.827 (4)	C8—H8B	0.9900
S3—C12	1.843 (4)	C9—H9A	0.9900
C1—H1A	0.9900	C9—H9B	0.9900
C1—H1B	0.9900	C9—C10	1.529 (5)
C1—C2	1.522 (6)	C10—H10A	0.9900
C2—H2A	0.9900	C10—H10B	0.9900
C2—H2B	0.9900	C10—C11	1.521 (5)
C2—C3	1.521 (6)	C11—H11A	0.9900
C3—H3A	0.9900	C11—H11B	0.9900
C3—H3B	0.9900	C11—C12	1.537 (5)
C3—C4	1.533 (5)	C12—H12A	0.9900
C4—H4A	0.9900	C12—H12B	0.9900
C4—H4B	0.9900		
			
Cl1—Ir1—Cl2	90.39 (3)	S2—C5—H5A	110.3
Cl1—Ir1—Cl3	177.35 (3)	S2—C5—H5B	110.3
Cl3—Ir1—Cl2	89.63 (3)	H5A—C5—H5B	108.6
S1—Ir1—Cl1	90.37 (3)	C6—C5—S2	107.0 (2)
S1—Ir1—Cl2	90.41 (3)	C6—C5—H5A	110.3
S1—Ir1—Cl3	92.28 (3)	C6—C5—H5B	110.3
S1—Ir1—S3	176.44 (3)	C5—C6—H6A	110.2
S2—Ir1—Cl1	91.09 (3)	C5—C6—H6B	110.2
S2—Ir1—Cl2	178.15 (3)	H6A—C6—H6B	108.5
S2—Ir1—Cl3	88.85 (3)	C7—C6—C5	107.4 (3)
S2—Ir1—S1	90.70 (3)	C7—C6—H6A	110.2
S2—Ir1—S3	92.61 (3)	C7—C6—H6B	110.2
S3—Ir1—Cl1	90.88 (3)	C6—C7—H7A	110.6
S3—Ir1—Cl2	86.25 (3)	C6—C7—H7B	110.6
S3—Ir1—Cl3	86.47 (3)	H7A—C7—H7B	108.8
C1—S1—Ir1	109.15 (13)	C8—C7—C6	105.5 (3)
C4—S1—Ir1	110.23 (12)	C8—C7—H7A	110.6
C4—S1—C1	93.79 (17)	C8—C7—H7B	110.6
C5—S2—Ir1	112.32 (13)	S2—C8—H8A	110.4
C8—S2—Ir1	110.12 (13)	S2—C8—H8B	110.4
C8—S2—C5	92.79 (17)	C7—C8—S2	106.6 (2)
C9—S3—Ir1	113.33 (12)	C7—C8—H8A	110.4
C9—S3—C12	93.79 (16)	C7—C8—H8B	110.4
C12—S3—Ir1	109.93 (12)	H8A—C8—H8B	108.6
S1—C1—H1A	110.3	S3—C9—H9A	110.9
S1—C1—H1B	110.3	S3—C9—H9B	110.9
H1A—C1—H1B	108.6	H9A—C9—H9B	108.9
C2—C1—S1	106.9 (3)	C10—C9—S3	104.2 (2)
C2—C1—H1A	110.3	C10—C9—H9A	110.9
C2—C1—H1B	110.3	C10—C9—H9B	110.9
C1—C2—H2A	110.4	C9—C10—H10A	110.7
C1—C2—H2B	110.4	C9—C10—H10B	110.7
H2A—C2—H2B	108.6	H10A—C10—H10B	108.8
C3—C2—C1	106.7 (3)	C11—C10—C9	105.5 (3)
C3—C2—H2A	110.4	C11—C10—H10A	110.7
C3—C2—H2B	110.4	C11—C10—H10B	110.7
C2—C3—H3A	110.5	C10—C11—H11A	110.4
C2—C3—H3B	110.5	C10—C11—H11B	110.4
C2—C3—C4	106.1 (3)	C10—C11—C12	106.8 (3)
H3A—C3—H3B	108.7	H11A—C11—H11B	108.6
C4—C3—H3A	110.5	C12—C11—H11A	110.4
C4—C3—H3B	110.5	C12—C11—H11B	110.4
S1—C4—H4A	110.8	S3—C12—H12A	110.4
S1—C4—H4B	110.8	S3—C12—H12B	110.4
C3—C4—S1	104.6 (2)	C11—C12—S3	106.5 (2)
C3—C4—H4A	110.8	C11—C12—H12A	110.4
C3—C4—H4B	110.8	C11—C12—H12B	110.4
H4A—C4—H4B	108.9	H12A—C12—H12B	108.6
			
Ir1—S1—C1—C2	106.1 (3)	C2—C3—C4—S1	43.0 (4)
Ir1—S1—C4—C3	−132.6 (2)	C4—S1—C1—C2	−6.9 (3)
Ir1—S2—C5—C6	106.8 (2)	C5—S2—C8—C7	−20.6 (3)
Ir1—S2—C8—C7	−135.6 (2)	C5—C6—C7—C8	−48.0 (4)
Ir1—S3—C9—C10	−138.6 (2)	C6—C7—C8—S2	42.1 (3)
Ir1—S3—C12—C11	114.0 (2)	C8—S2—C5—C6	−6.3 (3)
S1—C1—C2—C3	33.0 (4)	C9—S3—C12—C11	−2.5 (3)
S2—C5—C6—C7	31.9 (4)	C9—C10—C11—C12	−49.9 (4)
S3—C9—C10—C11	46.4 (3)	C10—C11—C12—S3	29.9 (4)
C1—S1—C4—C3	−20.5 (3)	C12—S3—C9—C10	−25.0 (3)
C1—C2—C3—C4	−49.8 (4)		
